# The rs12617336 and rs17574 Dipeptidyl Peptidase-4 Polymorphisms Are Associated With Hypoalphalipoproteinemia and Dipeptidyl Peptidase-4 Serum Levels: A Case-Control Study of the Genetics of Atherosclerotic Disease (GEA) Cohort

**DOI:** 10.3389/fgene.2021.592646

**Published:** 2021-06-11

**Authors:** Gilberto Vargas-Alarcón, María del Carmen González-Salazar, Christian Vázquez-Vázquez, Adrián Hernández-Díaz Couder, Fausto Sánchez-Muñoz, Juan Reyes-Barrera, Sergio A. Criales-Vera, Marco Sánchez-Guerra, Citlalli Osorio-Yáñez, Rosalinda Posadas-Sánchez

**Affiliations:** ^1^Departamento de Biología Molecular, Instituto Nacional de Cardiología Ignacio Chávez, Ciudad de Mexico, Mexico; ^2^Departamento de Endocrinologia, Instituto Nacional de Cardiología Ignacio Chávez, Ciudad de Mexico, Mexico; ^3^Departamento de Inmunologia, Instituto Nacional de Cardiología Ignacio Chávez, Ciudad de Mexico, Mexico; ^4^Departamento de Tomografía, Instituto Nacional de Cardiología Ignacio Chávez, Ciudad de Mexico, Mexico; ^5^Departamento de Neurobiologia del Desarrollo, Instituto Nacional de Perinatologia, Ciudad de Mexico, Mexico; ^6^Instituto de Investigaciones Biomédicas, Universidad Nacional Autónoma de Mexico, Ciudad de Mexico, Mexico

**Keywords:** Dipeptidyl peptidase-4, hypoalphalipoproteinemia, insulin resistance, hyperinsulinemia, polymorphism, DPP4 serum levels

## Abstract

Dipeptidyl peptidase-4 (DPP4) can influence lipid homeostasis and atherosclerosis progression. We aimed to assess the association of *DPP4* gene polymorphisms with hypoalphalipoproteinemia and DPP4 serum levels, in a cohort of Mexican individuals. Five *DPP4* polymorphisms (rs12617336, rs12617656, rs1558957, and rs3788979, and rs17574) were genotyped in 748 participants with and 745 without hypoalphalipoproteinemia. The associations were evaluated using logistic regression analyses. Under inheritance models adjusted for confounding variables, the rs12617336 (*OR* = 0.22, *P*_heterozygote_ = 0.001) and rs17574 (*OR* = 0.78, *P*_additive_ = 0.022; *OR* = 0.73, *P*_dominant_ = 0.012; *OR* = 0.73, *P*_heterozygote_ = 0.017; *OR* = 0.72, *P*_codominant__1_ = 0.014) minor alleles were associated with a low risk of hypoalphalipoproteinemia. After the correction for multiple comparisons, the associations were marginal except the association of the rs12617336 that remaining significant. Additionally, both *DPP4* minor alleles were associated with protection for the presence of insulin resistance (IR) (*OR* = 0.17, *P*_heterozygote_ = 0.019 for rs12617336 and *OR* = 0.75, *P*_additive_ = 0.049 for rs17574). The rs12617336 minor allele was also associated with a low risk of hyperinsulinemia (*OR* = 0.11, *P*_heterozygote_ = 0.006). Differences in DPP4 levels were observed in individuals with rs17574 genotypes, the rs17574 *GG* genotype individuals had the lowest levels. Our data suggest that rs12617336 and rs17574 *DPP4* minor alleles could be envisaged as protective genetic markers for hypoalphalipoproteinemia, IR, and hyperinsulinemia. The rs17574 *GG* genotype was associated with the lowest DPP4 levels.

## Introduction

Hyperglycemia, insulin resistance (IR), dyslipidemia, oxidative stress, and inflammation are well-documented risk factors for subclinical atherosclerosis (SA) and cardiovascular disease (CVD). Atherogenic dyslipidemia is characterized by an increase in triglyceride-rich lipoproteins, low high-density lipoprotein (HDL) cholesterol (HDL-C) levels, accumulation of lipoprotein remnants, and dense low-density lipoprotein (LDL) particles (Chapman et al., 2011; Ponte-Negretti et al., 2017). According to the results of the National Health and Nutrition Survey 2012, hypoalphalipoproteinemia and elevated LDL-cholesterol (LDL-C) are the most prevalent dyslipidemias among Mexican adults (Hernández-Alcaraz et al., 2020). Decreased levels of serum HDL-C (<40 mg/dl in men and <50 mg/dl in women) are a very significant independent risk factor for CVD (Ingelsson et al., 2007; Goff et al., 2014). Some genetic variants in different genes such as lipoprotein lipase, cholesteryl ester transfer protein, and ATP binding cassette transporter A-I have been associated with low HDL-C levels in patients with CVD (Brousseau et al., 2001, 2002, 2004).

Dipeptidyl peptidase-4 (DPP4, also known as CD26) is a multifunctional protein expressed in two molecular forms: a soluble form circulating in the plasma, and a membrane-anchored form. Both forms have peptidase activity and cleave a wide variety of substrates, such as incretins (GLP-1-glucagon-like peptide-1, and GIP-glucose-dependent insulinotropic polypeptide), chemokines, growth factors, and regulatory peptides (Mulvihill and Drucker, 2014). Previous studies have shown the participation of DPP4 in the pathogenesis of hyperglycemia, IR, dyslipidemia, oxidative stress, inflammation (Zhao et al., 2006; Lamers et al., 2011; Zheng et al., 2014; Zheng T. et al., 2015), fatty liver (FL) (Baumeier et al., 2017) and SA (Zheng T. P. et al., 2015). Moreover, DPP4 is an adipokine with increased expression in obesity (Lamers et al., 2011; Sell et al., 2013), FL (Miyazaki et al., 2012), and type 2 diabetes mellitus (T2DM) (Zhong et al., 2015), abnormalities that coexist with chronic inflammation. Considering the important role of the DPP4 in glucose metabolism, several DPP4-inhibitors have been used for T2DM treatment (Rabizadeh et al., 2020). Several polymorphisms have been described in the *DPP4* gene (located in region 2q24.3), some of which have been associated with rheumatoid arthritis (Jiang et al., 2014), T2DM, (Ahmed et al., 2016) and myocardial infarction in patients with coronary artery disease (CAD) (Aghili et al., 2012). In the same way, the DPP4 rs4664443 polymorphism was associated with different concentrations of DPP4 and apolipoprotein B (apoB) (Bailey et al., 2014; Ahmed et al., 2016). Considering the high prevalence of obesity and comorbidities such as T2DM (Rtveladze et al., 2014) and hypoalphalipoproteinemia in the Mexican population (Hernández-Alcaraz et al., 2020) and that the expression of DPP4 is increased in these abnormalities, the present study aimed to evaluate the association of the *DPP4* polymorphisms with the presence of hypoalphalipoproteinemia and with DPP4 levels in a cohort of Mexican individuals. Based on a functional prediction analysis, we decide to study 5 *DPP4* polymorphisms (rs12617336, rs12617656, rs1558957, rs3788979, and rs17574) with possible functional consequences and/or with minor allele frequency >5%.

## Methods

### Study Population

#### Ethics Statements

The study was conducted following the Declaration of Helsinki, and was approved by the Ethics Committee of the Instituto Nacional de Cardiología Ignacio Chávez (Project number 18-1082). All participants gave their written informed consent for inclusion before they participated in the study.

#### Cohort Description

All the participants were selected from the GEA Mexican study. This project was designed to examine the premature CAD (pCAD) genetic bases and the association with emerging and traditional cardiovascular risk factors in the Mexican population. All study subjects were not blood-related and were Mexican mestizos defined as a person who was born in Mexico and whose last two ascending generations were also born in Mexico. This report is a cross-sectional analysis of the baseline evaluation of the GEA study control group; this group includes 1,493 individuals without either pCAD personal or family history, enrolled from social service centers and blood banks.

#### Demographic, Clinic, and Biochemical Variables

In the whole sample, demographic, family and personal medical history, nutritional and smoking habits, physical activity, clinical, anthropometric, and biochemical variables and cardiovascular risk factors were evaluated and defined as previously described (Medina-Urrutia et al., 2015; Posadas-Sánchez et al., 2017a, b). Briefly, body mass index (BMI) was calculated as weight in kilograms divided by height in square meters, and obesity was defined when BMI ≥ 30 kg/m^2^. Waist circumference was measured using a glass fiber measuring tape in the middle point of the distance between the lower side of the waist and the iliac crest. Criteria from the American Heart Association National Heart, Lung, and Blood Institute Scientific Statement on the Metabolic Syndrome were used to define hypoalphalipoproteinemia and hypertriglyceridemia (Grundy et al., 2005). Non-HDL-C was defined when its values were >160 mg/dL. High apoB levels were considered when its values were >110 mg/dL (Grundy, 2002). LDL and HDL particle size were estimated with the LDL-C/apoB and HDL-C/apolipoprotein A (apoA1) ratios, respectively. HDL estimated diameter was calculated as described by Mazer et al. (2013). LDL pattern B was considered with an LDL-C/apoB ratio of 1.2, which corresponded to an LDL diameter of 25.5 nm, which is the cut-off value to distinguishing LDL pattern A from pattern B (Hirano et al., 2005). When individuals self-reported the current use of cigarettes, they were considered as current smoking. T2DM was defined by the American Diabetes Association criteria, with a fasting glucose ≥ 126 mg/dL, and was also considered when participants reported glucose-lowering treatment or a physician diagnosis of diabetes. Elevated high-sensitivity C-reactive protein (hsCRP) was considered when its values were ≥3 mg/L. Hyperuricemia was considered with a serum uric acid > 6.0 and >7.0 mg/dl for women and men (Ogbera and Azenabor, 2010), respectively. Hyperinsulinemia (HI) was defined when insulin concentration was ≥75th percentile (16.97 μU/mL in women and 15.20 μU/mL in men). IR was estimated using the homeostasis model assessment of insulin resistance (HOMA-IR). The presence of IR was considered when the HOMA-IR values were ≥75th percentile (3.66 in women and 3.38 in men). These cutoff points were obtained from a GEA Mexican study sample of 131 men and 185 women without obesity and with normal values of blood pressure, fasting glucose, and lipids. Visceral abdominal fat (VAF) area was quantified as described by Kvist et al. (1988) and the coronary artery calcification (CAC) score using the Agatston method (Mautner et al., 1994) after performed computed tomography of the chest and abdomen. Physical activity was measured using the Baecke questionnaire (Baecke et al., 1982). Total activity was obtained from the sum of the work exercise and leisure time activities. This questionnaire has been validated in adult populations and provides reliable information.

### Quantification of DPP4 Concentration

In all participants, DPP4 serum concentration was quantified using a Bioplex system (R&D Systems, Minneapolis, United States) according to the manufacturer’s instructions. The data were analyzed using the Bio-Plex Manager software. Results are expressed in ng/mL.

### Functional Prediction Analysis

To predict the possible effect of the *DPP4* polymorphisms, we used the following bioinformatics tools: FastSNP (Yuan et al., 2006), SNP Function Prediction^1^, Human-transcriptome Database for Alternative Splicing^2^, Splice Port: An Interactive Splice Site Analysis Tool^3^, ESE finder^4^, HSF^5^, and SNPs3D^6^. We used default parameters to run all bioinformatics tools.

### Genotyping

DNA isolation was performed according to the method proposed by Lahiri and Numberger (Lahiri and Nurnberger, 1991). The possible functional effect of the *DPP4* polymorphisms was defined using bioinformatics tools. For the analysis, we selected five *DPP4* gene polymorphisms with minor allele frequencies > 5% and/or possible functional consequences. Using 5′ exonuclease TaqMan genotyping assays, the rs12617336, rs12617656, rs1558957, rs3788979, and rs17574 *DPP4* polymorphisms were genotyped on an ABI Prism 7900HT Fast Real-Time PCR system (Applied Biosystems, Foster City, CA).

### Statistical Analysis

Data are expressed as frequencies, median (interquartile range), or mean ± standard deviation, as appropriate. Either Mann–Whitney *U* or Student’s *t*-test was used for continuous variable comparisons, while the chi-squared test was employed for categorical variable comparisons. The frequencies of alleles and genotypes were determined by direct counting. We used the chi-squared test to determine Hardy-Weinberg’s equilibrium. DPP4 serum concentration comparisons were evaluated by the Mann-Whitney *U*-test or Kruskal Wallis test, as appropriate. We used logistic regression analysis (adjusted for confounding variables as appropriate) to test for the relation of studied polymorphisms with hypoalphalipoproteinemia, and cardiovascular risk factors. The association analyses were made under the following models: additive (major allele homozygotes vs. heterozygotes vs. minor allele homozygotes), co-dominant (major allele homozygotes vs. heterozygotes and major allele homozygotes vs. minor allele homozygotes), dominant (major allele homozygotes vs. heterozygotes + minor allele homozygotes), heterozygous (heterozygotes vs. major allele homozygotes + minor allele homozygotes), and recessive (major allele homozygotes + heterozygotes vs. minor allele homozygotes). Hosmer–Lemeshow Goodness of Fit test was performed for each multiple logistic model. The *p*-value was corrected by multiple comparisons and our threshold for significance was 0.01. All analyses were performed using SPSS software v15.0 (SPSS Chicago, IL).

## Results

### Study Sample Characteristics

One thousand four hundred ninety-three individuals belonging to the GEA Mexican study control group were included in the present analysis. According to the HDL-C levels, 748 were hypoalphalipoproteinemia individuals (<40 mg/dl in men and <50 mg/dl in women), while 745 were considered non-hypoalphalipoproteinemia individuals. Table 1 shows demographic, lifestyle, clinical, and biochemical characteristics as well as the tomographic data and study population genotypes. Age, percentage of male, waist circumference, and the presence of T2DM were similar in both groups. Compared with non-hypoalphalipoproteinemia individuals, BMI, triglycerides and apoB levels, VAF, and the prevalence of HI, IR, hyperuricemia, hsCRP concentration, high non-HDL-C levels, high apoB levels, LDL pattern B, and current smoking were significantly higher in hypoalphalipoproteinemia subjects. On the contrary, HDL-C, apoA1, and DPP4 concentrations, HDL diameter, HDL and LDL size, physical activity, and the percentage of rs12617336 *GC* and rs17574*GG* genotypes were higher in non-hypoalphalipoproteinemia subjects than in hypoalphalipoproteinemia ones (Table 1).

**TABLE 1:** Demographic, lifestyle, clinical characteristics, lipid profile, tomographic data, and genotypes in the studied groups.

	Hypoalphalipoproteinemia		
	No (*n* = 745)	Yes (*n* = 748)	*P-value
**Demographic**			
Age (years)	54 ± 9	52 ± 9	0.088
Sex (% male)	50.7	49.7	0.368
**Clinical characteristics and coronary risk factors**			
Body mass index (kg/m^2^)	27.1 (24.6–29.9)	28.6 (26.4–31.4)	<0.001
Waist circumference (cm)	96.6 ± 11.3	96.5 ± 10.7	0.879
Type 2 diabetes mellitus (%)	46.2	53.8	0.283
Hyperinsulinemia (%)	45.7	63.5	<0.001
Insulin resistance (%)	47.5	66.7	<0.001
Hyperuricemia (%)	19.1	25.5	0.003
High sensitivity C reactive protein ≥ 3mg/L (%)	23.3	30.8	0.001
Non-HDL-cholesterol > 160mg/dL (%)	28.9	34.3	0.026
High apolipoprotein B (%)	25.9	34.3	<0.001
**Lipid profile and DPP4 concentration**			
HDL-cholesterol (mg/dL)	54 (47–61)	36 (32–41)	<0.001
Triglycerides (mg/dL)	126 (95–165)	179 (132–236)	<0.001
Apolipoprotein A1 (mg/dL)	149 (129–169)	120 (105–138)	<0.001
Apolipoprotein B (mg/dL)	92 (75–111)	98 (79–117)	<0.001
Estimated HDL size	0.37 (0.33–0.42)	0.30 (0.26–0.35)	<0.001
Estimated HDL diameter	9.20 (8.70–9.84)	8.37 (7.91–8.94)	<0.001
Estimated LDL size	1.26 (1.13–1.43)	1.16 (1.04–1.30)	<0.001
Pattern B (%)	38.0	55.9	<0.001
DPP4 serum concentration (ng/mL)	125.2 (98.4–153.7)	117.8 (94.6–148.9)	0.008
**Tomography**			
Visceral abdominal fat (cm^2^)	134 (98–186)	159 (124–201)	<0.001
**Lifestyle**			
Current smoking habit (%)	19.5	25.2	0.009
Physical activity	8.0 (7.1–8.9)	7.8 (6.9–8.6)	0.002
**DPP4 genotype (%)**			
rs12617336 (*GG/GC/CC*)	96.5/3.5/0	98.8/1.2/0	0.003
rs12617656 (*CC/CT/TT*)	38.8/47.4/13.8	36.7/46.5/54.8	0.276
rs1558957 (*CC/CT/TT*)	29.0/51.5/19.5	31.3/49.5/19.2	0.603
rs3788979 (*CC/CT/TT*)	50.5/40.1/9.5	46.5/43.8/9.8	0.286
rs17574 (*AA/AG/GG*)	62.6/33.5/3.9	71.1/25.5/3.3	0.002

Data are shown as mean ± standard deviation, median (interquartile range) or percentage. *Student’s t-test, Mann Whitney’s U-test, or Chi-square test. HDL, high-density lipoprotein; LDL, low-density lipoprotein.

### Association of Polymorphisms With Hypoalphalipoproteinemia

**FIGURE 1:**
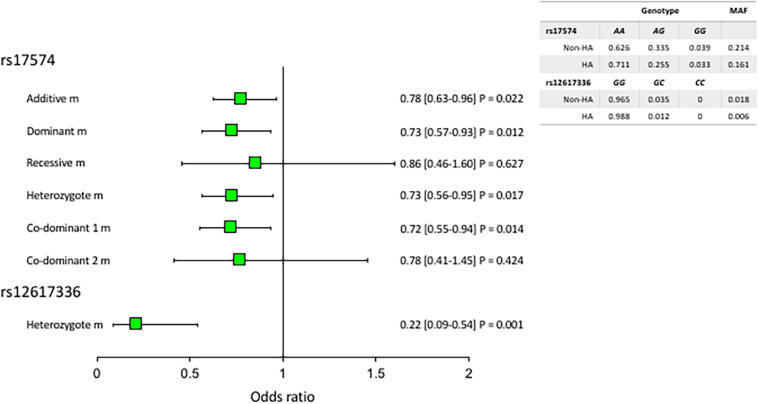
Association of *DPP4* polymorphisms with hypoalphalipoproteinemia. MAF, minor allele frequency. All models were adjusted by age, sex, BMI, current smoking, physical activity, visceral abdominal fat, and triglycerides levels. The reference genotype was *GG* for rs12617336 and *AA* for rs17574. HA: hypoalphalipoproteinemia; Non-HA: non-hypoalphalipoproteinemia.

All the polymorphisms were in Hardy-Weinberg equilibrium. Figure 1 shows the associated *DPP4* polymorphisms with hypoalphalipoproteinemia. For this analysis, we adjusted each model for age, sex, BMI, VAF, current smoking, physical activity, and triglycerides levels. Under the heterozygote model, the rs12617336 minor allele (0.22 [0.09–0.54] *P* = 0.001) was significantly associated with a low risk of hypoalphalipoproteinemia. Additionally, the rs17574 minor allele was also associated with protection for the presence of hypoalphalipoproteinemia, under additive (0.78 [0.63–0.96] *P* = 0.022) dominant (0.73 [0.57–0.93] *P* = 0.012), heterozygote (0.73 [0.56–0.95] *P* = 0.017) and co-dominant 1 (0.72 [0.55–0.94] *P* = 0.014) models. After the correction by multiple comparisons, the associations were marginal except the association of the rs12617336 that remaining significant. According to the Hosmer-Lemeshow criteria, the most appropriate inheritance models were dominant and co-dominant 1.

### Cardiovascular Risk Factors and Their Relation With rs12617336 and rs17574 DPP4 Polymorphisms

Table 2 shows the associations of *DPP4* rs12617336 and rs17574 polymorphisms with cardiovascular risk factors in individuals with and without hypoalphalipoproteinemia. In individuals with hypoalphalipoproteinemia, under heterozygote model, the rs12617336 minor allele was associated with lower risk of the presence of IR (*OR* = 0.17, *P* = 0.019) and HI (*OR* = 0.11, *P* = 0.006), and with 6 times higher risk of the presence of hyperuricemia (*OR* = 6.30, *P* = 0.012). On the other hand, in individuals without hypoalphalipoproteinemia, the rs17574 minor allele was related with 25% lower risk of IR (*OR* = 0.75, *P*_additive_ = 0.049), approximately 40% higher risk of high non-HDL-C (*OR* = 1.47, *P*_heterozygote_ = 0.023; *OR* = 1.45, *P*_co__–__dominant__1_ = 0.033) and high apoB (*OR* = 1.44, *P*_heterozygote_ = 0.043; Table 2).

**TABLE 2:** *DDP4* gene polymorphisms association with cardiovascular risk factors.

Polymorphism	Genotype frequency			MAF	Model	OR (95% CI)	*P*
**Hypoalphalipoproteinemia (*n* = 748)**							

**rs12617336**	***GG***	***GC***	***CC***				

**Insulin resistance**							
No (*n* = 247)	0.980	0.020	0	0.010	Heterozygote	0.17 (0.04–0.75)	0.019
Yes (*n* = 501)	0.992	0.008	0	0.004			
**Hyperinsulinemia**							
No (*n* = 272)	0.978	0.022	0	0.011	Heterozygote	0.11 (0.02–0.52)	0.006
Yes (*n* = 476)	0.994	0.006	0	0.003			
**Hyperuricemia**							
No (*n* = 557)	0.995	0.005	0	0.003	Heterozygote	6.30 (1.51–26.34)	0.012
Yes (*n* = 191)	0.969	0.031	0	0.016			

**Non-hypoalphalipoproteinemia (*n* = 745)**							

**rs17574**	***AA***	***AG***	***GG***				

**Insulin resistance**							
No (*n* = 389)	0.588	0.366	0.046	0.229	Additive	0.75 (0.56–0.99)	0.049
Yes (*n* = 356)	0.668	0.301	0.031	0.181			
**Non-HDL-C > 160mg/dL**							
No (*n* = 530)	0.643	0.314	0.043	0.200	Heterozygote	1.47 (1.06–2.07)	0.023
Yes (*n* = 215)	0.584	0.388	0.028	0.221	Co-dominant 1	1.45 (1.03–2.03)	0.033
**High apolipoprotein B**							
No (*n* = 551)	0.636	0.320	0.044	0.203	Heterozygote	1.44 (1.01–1.04)	0.043
Yes (*n* = 194)	0.589	0.385	0.026	0.219			

*MAF, minor allele frequency; OR, odds ratio; 95% CI, 95% confidence interval; p75, percentile 75. All models were adjusted by age, gender, and body mass index.*

### Relationship Between DPP4 Genotypes and DPP4 Serum Concentrations

**FIGURE 2:**
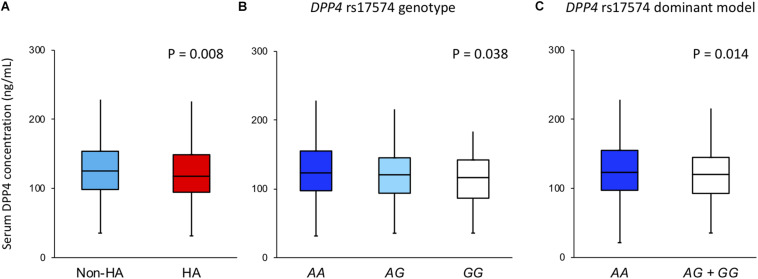
DPP4 concentration in the whole sample stratified by **(A)** in non-hypoalphalipoproteinemia and hypoalphalipoproteinemia groups; **(B)** in rs17574 genotypes; **(C)** in non-carries (*AA*) and carriers (*AG* + *GG*) of rs17574 minor allele. HA: Hypoalphalipoproteinemia; Non-HA: non-hypoalphalipoproteinemia.

Figure 2A shows that DPP4 serum concentration was higher in the non-hypoalphalipoproteinemia subjects when compared with hypoalphalipoproteinemia participants (125.2 [98.4–153.7] ng/mL vs. 117.8 [94.6–148.9] ng/mL, *P* = 0.008, respectively). In the same way, the DPP4 concentration of the whole sample was analyzed stratifying for rs17574 *DPP4* genotypes. Carriers of the rs17574 *AA* genotype have the highest DPP4 serum concentration (123.3 [97.3–155.1] ng/mL) compared with *AG* (120.6 [93.7–145.2] ng/mL) and *GG* genotypes (116.5 [86.4–142.1] ng/mL, *P* = 0.038; Figure 2B), and compared to carriers of *G* allele (*AG* and *GG* genotypes) (120.3 [92.8–144] ng/mL, *P* = 0.014 vs. *AA* genotype; Figure 2C).

**FIGURE 3:**
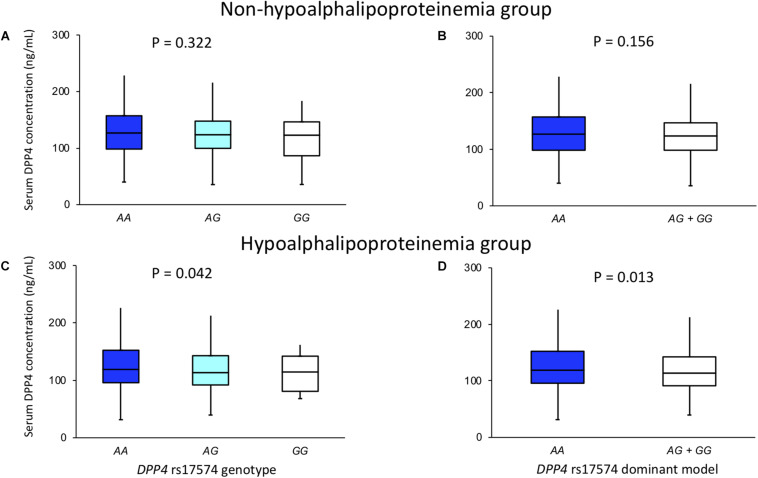
DPP4 concentration in non-hypoalphalipoproteinemia **(A,B)** and hypoalphalipoproteinemia **(C,D)** groups, stratified by rs17574 genotypes **(A,C)** and in non-carries (*AA*) and carriers (*AG* + *GG*) of rs17574 minor allele **(B,D)**.

The DPP4 serum concentration and its association with rs17574 genotypes were evaluated separately in individuals with and without hypoalphalipoproteinemia (Figure 3). In individuals, without hypoalphalipoproteinemia, no significant differences were observed (Figures 3A,B). On the other hand, in individuals with hypoalphalipoproteinemia, subjects with *AA* genotype had the highest DPP4 concentration (119 [95.9–152.4] ng/mL) compared with *AG* (113.5 [92.0–142.9] ng/mL) and *GG* genotypes (114.6 [80.9–142.1], *P* = 0.042; Figure 3C); also, when compared with carriers of *G* allele (*AG* and *GG* genotypes) (113.8 [91.4–142.5] ng/mL, *P* = 0.013 vs. *AA* genotype; Figure 3D). Due to the lack of the presence of the *CC* genotype of the rs12617336 polymorphism in our population, we were unable to assess the association of the genotypes of this polymorphism with DPP4 concentrations.

### Polymorphism Functional Prediction

Polymorphism functional prediction software results suggest that the rs12617336 and rs17574 have a possible functional effect. The variation in the rs12617336 polymorphism, located in the 3′-UTR region produces binding sites for some microRNAs such as microRNA-939, 708, 1244, and 1238. On the other hand, the rs17574 located in exon 2, affects the alternative splicing and modifies the affinity for the SF2ASF1 and SF2ASF2 proteins.

## Discussion

To the best of our knowledge, this report shows the association of rs12617336 and rs17574 *DPP4* minor alleles with a low risk of hypoalphalipoproteinemia for the first time. The minor alleles of these polymorphisms were also associated with a low risk of IR (rs12617336 and rs17574) and HI (rs12617336), high risk of hyperuricemia (rs12617336), non-HDL-C > 160 mg/dL (rs17574), and apoB >110 mg/dL (rs17574). On the other hand, individuals without hypoalphalipoproteinemia presented higher DPP4 levels when compared to individuals with hypoalphalipoproteinemia. In the whole sample, carriers of rs17574 *AA* genotype showed the highest DPP4 levels. Thus, individuals with the *G* allele presented low levels of DPP4, which agrees with the protective effect of this allele.

Adipocytes secrete several active proteins called adipokines and it is well-known that the deregulation of their secretion is associated with metabolic diseases (Wellen and Hotamisligil, 2005). DPP4 is expressed in adipocytes, and in patients with obesity, its expression and activity are elevated (Stengel et al., 2014). DPP4 plasma levels are related to several markers of obesity, such as BMI, waist circumference, plasma triglyceride levels, leptin concentration, and fat cell volume (Lamers et al., 2011). In this report, the rs12617336 and rs17574 minor alleles were associated with a 20–25% risk reduction for the presence of hypoalphalipoproteinemia, a metabolic abnormality that often coexists with obesity. Neither of the minor alleles of rs17574 or rs12617336 has been previously associated with hypoalphalipoproteinemia. Turcot et al. reported that individuals with rs17574 *GG* genotype had higher levels of methylation in the promoter CpG islands in the visceral adipose tissue (Turcot et al., 2011). They also showed a positive relationship between methylation levels and HDL-C concentration (Turcot et al., 2011). Additionally, using informatics tools, important possible functional effects were defined for the two polymorphisms associated with hypoalphalipoproteinemia. The rs17574 polymorphism, that we report associated with a low risk of hypoalphalipoproteinemia, is located in exon 2 of the gene; the computer analysis that we carried out establishes that the *G* allele in this position can affect the efficiency of cutting and splicing, modifying the binding affinity for splicing factors SF2ASF1 and SF2ASF2. This suggests that this polymorphism could be related to the production of DPP4 isoforms with altered activity. Previously, various forms of DPP4 were detected in human and placental plasma (Puschel et al., 1982; Krepela et al., 1983), as well as in lung cancer and normal tissues (Sedo et al., 1991, 1998). DPP4 isoforms with altered activity could be involved in the development of hypoalphalipoproteinemia. On the other hand, the rs12617336 *G* allele produces a binding site for the miRNA-939, miRNA-708, and miRNA-1244, whereas the *C* allele produces a binding site for the miRNA-1238. It has also been reported that HDLs transport miRNAs (Ren et al., 2013; Wagner et al., 2013), specifically, it has been reported that the miRNA-223 regulates crucial pathways for maintaining cardiovascular homeostasis (Vickers et al., 2011) and in consequence, protects against atherosclerosis by modulating HDL cholesterol efflux capacity and lipid metabolism (Vickers et al., 2014; Ono, 2016). These data agree with our results showing that the rs12617336 and rs17574 minor alleles are associated with a reduced risk for hypoalphalipoproteinemia. Our findings suggest that rs12617336 *C* and rs17574 *G* alleles could be considered as potential genetic markers for hypoalphalipoproteinemia in our population.

DPP4 enzymatic activity inactivates GLP-1, a hormone that regulates postprandial insulin secretion (Gorrell, 2005). Therefore, DPP4 increased level and/or activity may impair insulin sensitivity causing IR and HI. It has been reported that DPP4 serum levels were higher in individuals with IR as compared to insulin-sensitive subjects matched for BMI (Sell et al., 2013). Moreover, in T2DM it has been shown that increased DPP4 activity is positively associated with IR with a significant increase in IR with rising DPP4 activity quartiles (Zheng T. P. et al., 2015). In the same way, it has been reported that increase DPP4 mRNA levels in the liver of patients with non-alcoholic FL disease correlate with IR (Miyazaki et al., 2012). It has also been reported that DPP4 expression correlates with the amount of VAF, adipocyte size, and inflammation (Sell et al., 2013). Macrophages and dendritic cells present in VAF exhibit an increased DPP4 expression in response to an obese state or inflammation (Zhong et al., 2013). The best-known non-catalytic function DPP4 exerts is the co-stimulation of T cells through interaction with adenosine deaminase (Kameoka et al., 1993) and modulation of the function of antigen-presenting cells (Zhong et al., 2015). This suggests that DPP4 could have an important participation in the chronic low-grade inflammation present in dyslipidemias, T2DM, IR, obesity, and atherosclerosis. When chronic inflammation is present in the liver and adipose tissue, the activated resident macrophages release pro-inflammatory cytokines; by a direct interaction between inflammatory pathways and insulin signaling, these cytokines can cause IR (de Luca and Olefsky, 2008), and in consequently HI. These data support our findings that showed an association between the rs17574 and rs12617336 minor alleles with a low risk for IR and HI (rs12617336). In our study, the carriers of the protective genotype rs17574 *GG* showed the lowest levels of serum DPP4. In this case, the lower DPP4 levels in these individuals would result in higher incretin levels and, in consequence, in insulin sensitivity.

Of the five *DPP4* polymorphisms included in the present study, three of them have previously been associated with serum lipid levels (Bouchard et al., 2009; Xing et al., 2016) and apoB levels (Bailey et al., 2014). Among T2DM individuals, dyslipidemia is a major risk factor for CVD (Mooradian, 2009). ApoB plasma levels, the principal protein component of very-low-density lipoprotein (VLDL) remnant, VLDL, and LDL particles, represent the total atherogenic lipoprotein particles in circulation and correlates with the concentration of non-HDL-C (Pischon et al., 2005). In South Asians, Bailey et al. (2014) identified and replicated the association between variation at the rs4664443 *DPP4* polymorphism and apoB levels, the association that was observed also in European individuals with BMI < 25 kg/m^2^. On the other hand, the *DPP4* rs1558957 polymorphism has been previously associated with high total cholesterol, HDL-C, LDL-C, and triglycerides plasma concentrations in a study of obese individuals of European ancestry, although these results were inconsistent (Bouchard et al., 2009). These previous reports support our findings. Here, we show an association between rs17574 minor allele and the presence of high apoB and non-HDL-C levels. It has been reported that the pharmacological inhibition of DPP4 is associated with lower total cholesterol, apoB, triglycerides, non-HDL-C, and VLDL levels (Tremblay et al., 2011; Monami et al., 2012; Kim et al., 2016).

Uric acid, the product of purine metabolism, is a natural antioxidant. However, it is well recognized that hyperuricemia is associated with metabolic diseases and endothelial dysfunction. We reported an association of the rs12617336 minor allele with a high risk of hyperuricemia. The relationship between the uric acid and DPP4 was established by Mohandas et al., who demonstrated that uric acid inhibits DPP4 activity when it is anchored to the membrane and that the inhibitory effect depends on the redox state of cells and the formation of intracellular triuret (Mohandas et al., 2014).

DPP4 is ubiquitously expressed on the surface of several cell types, however, it can also be found in circulation after it is shed from the membrane by proteolytic cleavage (Röhrborn et al., 2014). Increased DPP4 circulatory levels, in metabolic diseases, could be explained by an aberrant DPP4 shedding. It has been reported that in smooth muscle cells and adipocytes, hypoxia increased DPP4 shedding by matrix metalloproteases (Röhrborn et al., 2014). The constitutive shedding mechanism of DPP4 is varied with cell specificity as well as cells and tissue circumstances and occurs due to a complex interplay between different proteases in a cell-type-specific manner (Nargis and Chakrabarti, 2018). Our results showed that non-hypoalphalipoproteinemia subjects have significantly higher DPP4 concentrations. These findings appear to be contradictory to those expected. However, epigenetic modifications and changes at the DNA level are part of the complex mechanisms that modulate the production of several molecules, including DPP4. As previously mentioned, Turcot et al. in 2011 reported in premenopausal obese non-diabetic women that carriers of the rs17574 *GG* genotype have higher levels of methylation (Turcot et al., 2011). Our findings show that carriers of the rs17574 *GG* genotype have significantly lower concentrations of DPP4 compared to *AG* and *AA* genotypes. When stratified by the presence of hypoalphalipoproteinemia, the statistically significant difference was maintained in the subjects with hypoalphalipoproteinemia. Individuals with hypoalphalipoproteinemia, have a higher amount of VAF in comparison with non- hypoalphalipoproteinemia subjects (159 vs. 134 cm^2^, respectively, *p* < 0.001). The aforementioned suggests that in this group, the presence of higher VAF could be associated with an increase in the percentage of methylation in carriers of the rs17574 *GG* genotype, and thus, lead to lower expression of the DPP4 gene and, therefore, to lower levels in protein plasma.

The main strength of the present work is the inclusion of a large cohort of Mexican individuals, with and without hypoalphalipoproteinemia, who were broadly characterized from a tomographic, clinical, and biochemical point of view using standardized methods, which allowed adjustment of the results by an important number of potential confounders. Although the selection of the participants was not random, the studied subjects were not aware of being carriers of the variants of the *DPP4* gene studied, given this, it could be expected that the observed associations were similar to those of a sample random and therefore be applied to the general population. Despite these strengths, our study has some limitations. First, due to the cross-sectional nature of the study, we cannot make causal conclusions. Second, our results were not replicated in a second cohort of individuals with and without hypoalphalipoproteinemia. Thirdly, IR was not determined by the gold standard: the euglycemic clamp; nevertheless, insulin sensitivity can be accurately estimated by the HOMA-IR index that has proven to be a reliable measure (Bonora et al., 2000). Fourthly, our data may not apply to other ethnicities, considering that the GEA participants are exclusively Mexican-Mestizo subjects. The *DPP4* polymorphism associations detected in our study should be investigated in other populations to establish if they are shared with other ethnic groups or are specific to the Mexican population.

Our data suggest that rs12617336 and rs17574 *DPP4* minor alleles could be envisaged as protective genetic markers for hypoalphalipoproteinemia, IR, and hyperinsulinemia. The rs17574 *GG* genotype was associated with the lowest DPP4 levels. Considering that this is the first study to report the association of *DPP4* polymorphisms with the presence of hypoalphalipoproteinemia, studies in other populations are necessary to confirm our results.

## Data Availability Statement

The raw data supporting the conclusions of this article will be made available by the authors, without undue reservation.

## Ethics Statement

The studies involving human participants were reviewed and approved by the Ethics Committee of the Instituto Nacional de Cardiología Ignacio Chávez (Project No. 18-1082). The patients/participants provided their written informed consent to participate in this study.

## Author Contributions

RP-S and GV-A: conceptualization. FS-M, JR-B, CV-V and AH-D: methodology. CO-Y and MS-G: formal analysis. GV-A and RP-S: Resources. MG-S, CO-Y, and MS-G: visualization. SC-V and MG-S: supervision. MG-S, SC-V, GV-A, and RP-S: project administration. RP-S: Funding acquisition. MS-G, SC-V, JR-B, CV-V, AH-D, and FS-M: data curation. RP-S, GV-A, SC-V, and MG-S: writing—original draft preparation. GV-A and RP-S: writing—review and editing. All authors have contributed significantly, read and agreed to the published version of the manuscript.

## Conflict of Interest

The authors declare that the research was conducted in the absence of any commercial or financial relationships that could be construed as a potential conflict of interest.

## Footnotes

1
http://snpinfo.niehs.nih.gov/snpinfo/snpfunc.html

2 http://www.h-invitational.jp/h-dbas/

3 http://spliceport.cbcb.umd.edu/SplicingAnalyser.html

4 http://rulai.cshl.edu/cgi-bin/tools/ESE3/esefinder.cgi

5 http://www.umd.be/HSF/

6 http://www.snps3d.org/
